# Analytical Methods in Untargeted Metabolomics: State of the Art in 2015

**DOI:** 10.3389/fbioe.2015.00023

**Published:** 2015-03-05

**Authors:** Arnald Alonso, Sara Marsal, Antonio Julià

**Affiliations:** ^1^Rheumatology Research Group, Vall d’Hebron Research Institute, Barcelona, Spain; ^2^Department of Automatic Control (ESAII), Polytechnic University of Catalonia, Barcelona, Spain

**Keywords:** metabolomics, nuclear magnetic resonance, mass spectrometry, untargeted, spectral processing, data analysis, pathway analysis, integration

## Abstract

Metabolomics comprises the methods and techniques that are used to measure the small molecule composition of biofluids and tissues, and is actually one of the most rapidly evolving research fields. The determination of the metabolomic profile – the metabolome – has multiple applications in many biological sciences, including the developing of new diagnostic tools in medicine. Recent technological advances in nuclear magnetic resonance and mass spectrometry are significantly improving our capacity to obtain more data from each biological sample. Consequently, there is a need for fast and accurate statistical and bioinformatic tools that can deal with the complexity and volume of the data generated in metabolomic studies. In this review, we provide an update of the most commonly used analytical methods in metabolomics, starting from raw data processing and ending with pathway analysis and biomarker identification. Finally, the integration of metabolomic profiles with molecular data from other high-throughput biotechnologies is also reviewed.

## Introduction

Metabolomics is the study of the metabolite composition of a cell type, tissue, or biological fluid. The analysis of the complete set of metabolites – the metabolome – has been present in biological research for more than a decade (Patti et al., 2012). However, major recent advances in the technologies used to extract and analyze this type of molecular data have revolutionized its applicability in the analysis of organisms and relevant biological processes (Zhang et al., 2012). To date, metabolomics is envisaged as one of the major “omics” tools that will most contribute into challenging research objectives like the personalization of treatments in medical practice.

The metabolites are the intermediates or end products of multiple enzymatic reactions and therefore are the most informative proxies of the biochemical activity of an organism. The present technologies are allowing the study of tens to hundreds of metabolites in complex biological samples (Patti et al., 2012). One of the facts that is most contributing to the rapid growth of metabolomics is its wide range of applications. These applications cover diverse research areas like plant biology (Qi and Zhang, 2014), nutrition (Orešič, 2009; Gibbons et al., 2015), animal breeding (Kühn, 2012), drug discovery (Robertson and Frevert, 2013; Kell and Goodacre, 2014), and the study of human diseases (Kaddurah-Daouk et al., 2008; Mamas et al., 2011). The biomedical field is actually one of the most active areas of development in metabolomics, and includes the search for diagnostic and prognostic biomarkers as well as predictors of treatment response (Meyer et al., 2013; Armitage and Barbas, 2014; Julià et al., 2014). Also in this field, the use of metabolomics is helping to characterize the impact of key environmental factors on human health. In this area, one of the most promising applications is the characterization of gut–microbiota interactions in humans (Wikoff et al., 2009; Nicholson et al., 2012).

To date, the two main technical approaches for the generation of metabolomic data are nuclear magnetic resonance (NMR) and mass spectrometry (MS; Fuhrer and Zamboni, 2015). NMR is a fast and highly reproducible spectroscopic technique that is based on the energy absorption and re-emission of the atom nuclei due to variations in an external magnetic field (Bothwell and Griffin, 2011). Depending on the atom nuclei being targeted by the applied magnetic field, different types of metabolomic data are generated. However, in the analysis of samples of biological origin, hydrogen is the most commonly targeted nucleus (^1^H-NMR), due to its natural abundance in biological samples. Although less frequent, other atoms like carbon (^13^C-NMR) and phosphorus (^31^P NMR) are also targeted by NMR, providing additional information on specific metabolite types (Reo, 2002).

**Figure 1:**
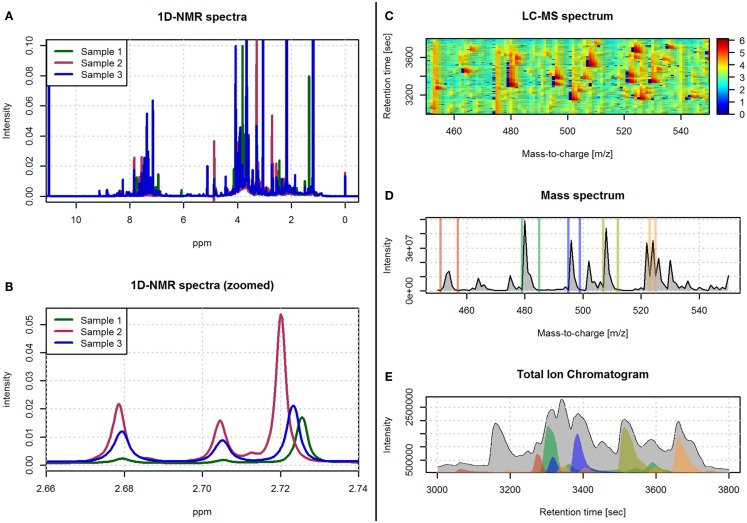
**Examples of spectra obtained with ^1^H-NMR and LC-MS technologies**. **(A)** An example of three spectra obtained with 1D ^1^H-NMR. **(B)** A zoomed view of the spectra in **(A)** in the 2.66–2.74 ppm range. **(C)** An example of a LC-MS spectrum with color-coded intensity and referred by the m/z and retention time axes. **(D)** The sum of the LC-MS spectrum across the m/z axis. **(E)** The total ion chromatogram (i.e., sum of the LC-MS spectrum across the retention time axis). The colored regions in **(E)** correspond to the sum of the LC-MS spectrum limited to the m/z ranges depicted with the same color in **(D)**.

The resulting spectral data in NMR not only allows the quantification of the concentration of metabolites but also provides information about its chemical structure. The spectral peak areas generated by each molecule are used as an indirect measure of the quantity of the metabolite in the sample, while the pattern of spectral peaks informing on the physical properties of the molecule is used to identify the type of metabolite. The spectral data obtained with NMR techniques can be referenced to one or two frequency axes. One dimensional NMR (1D-NMR) spectra are based on a single frequency axis, where the peaks of each molecule are placed within its resonant frequencies (Figure 1). 1D-NMR is the most commonly used method in high-throughput metabolomics studies. Conversely, two dimensional NMR (2D-NMR) spectra are based on two frequency axis, and its use is often restricted to the characterization of those compounds that cannot be identified with 1D-NMR spectra. The second dimension in 2D-NMR allows to separate otherwise overlapping spectral peaks and, therefore, gives additional and important information on the chemical properties of the metabolite (Ward et al., 2007). Although 2D-NMR generates a large number of different spectra, these can be globally classified into homonuclear (i.e., ^1^H–^1^H-NMR) and heteronuclear (i.e., ^1^H–^13^C or ^1^H–^15^N) spectra (Marion, 2013). There are also different pulse sequences used to generate the 2D-NMR spectra such as correlation spectrometry (COSY), total correlation spectroscopy (TOCSY), and nuclear Overhauser effect spectroscopy (NOESY). 1D- and 2D-NMR frequency axes are usually referenced by the chemical shift expressed in parts per million (ppm). The chemical shift is calculated as the difference between the resonance frequency and that of a reference substance, subsequently divided by the operating frequency of the spectrometer (Blümich and Callaghan, 1995).

Mass spectrometry is an analytical technique that acquires spectral data in the form of a mass-to-charge ratio (m/z) and a relative intensity of the measured compounds. For the spectrometer to generate the peaks signals for each metabolite, the biological sample first needs to be ionized. The resulting ionized compounds from each molecule will then generate different peak patterns that define the fingerprint of the original molecule. A wide range of instrumental and technical variants are currently available for MS spectrometry. These variants are mainly characterized by different ionization and mass selection methods (El-Aneed et al., 2009). In metabolomics, MS is generally preceded by a separation step. This step reduces the high complexity of the biological sample and allows the MS analysis of different sets of molecules at different times. Liquid and gas chromatography columns (LC and GC, respectively) are the most commonly used separation techniques (Theodoridis et al., 2011). This chromatographic separation technique is based on the interaction of the different metabolites in the sample with the adsorbent materials inside the chromatographic column. This way, metabolites with different chemical properties will require different amounts of time to pass through the column. The time that each metabolite requires, called retention time, is used together with the m/z MS values to generate the two axes of the LC-MS and GC-MS spectral data (Figure 1).

In the present review, we will describe the processing and analysis workflows that are commonly used in high-throughput untargeted metabolomic studies. Untargeted metabolomic studies are characterized by the simultaneous measurement of a large number of metabolites from each sample. This strategy, known as top-down strategy, avoids the need for a prior specific hypothesis on a particular set of metabolites and, instead, analyses the global metabolomic profile. Consequently, these studies are characterized by the generation of large amounts of data. This data is not only characterized by its volume but also by its complexity and, therefore, there is a need for high performance bioinformatic tools. Conversely, targeted metabolomic studies are hypothesis-driven experiments and are characterized by the measurement of predefined sets of metabolites with a high level of precision and accuracy. This low level of metabolite analysis is not in the scope of this review, and interested readers are referred to other excellent specific reviews (Roberts et al., 2012; Putri et al., 2013).

**Figure 2:**
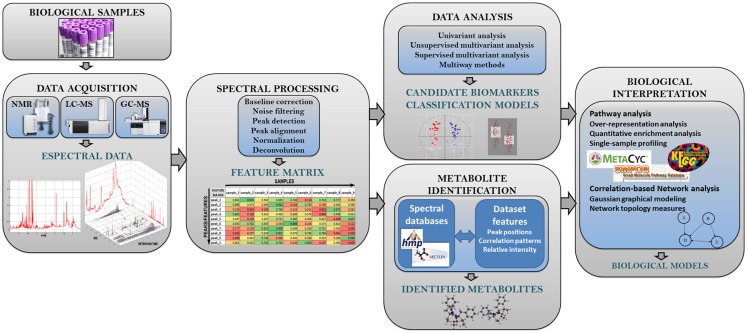
**Analysis workflow in untargeted metabolomic studies**. This figure shows the different steps of the metabolomic analysis pipeline.

In Figure 2, we show the typical methodological pipeline of an untargeted metabolomic study. This methodological pipeline starts with the processing of the spectral data to generate the sample metabolic information (i.e., metabolic features). The different methods available to process spectral data are revised in Section “Spectral Processing.” Together with metabolite-identification methods, spectral processing methods are highly dependent on the analytical technique used (e.g., NMR, LC-MS, or GC-MS). Once the complete set of metabolomic features has been generated, univariant and multivariant data analysis methods can be applied to investigate: (a) the general structure of the metabolomics data in the dataset and (b) how the different metabolic features are related with the phenotypic data associated with the samples. These analysis methods are reviewed in Section “Data Analysis.” The analysis of metabolomic data can often be used to build models that attempt to describe the observed data. Section “Biomarker Discovery in Metabolomics” of the present review describes the different strategies for assessing the performance of these models. In Section “Metabolite Identification and Spectral Databases,” we address the important technical issue that is the identification of the metabolites underlying the observed metabolic features (i.e., peak areas and spectral bins). The bioinformatic methods that are actually available for the integration of metabolomic data according with biological knowledge are reviewed in Section “Pathway and Network Analysis of Metabolomic Data.” Finally, the different methodologies that allow the integration of metabolomics data with other omics data (e.g., genomics or transcriptomics) are reviewed in Section “Integration of Omics Data.” Table 1 shows a list of the freely available tools that are most commonly used in metabolomic analysis. These tools provide different methodological options for spectral processing, data analysis, or pathway analysis.

**Table 1:** **List of tools available for metabolomics spectral processing and data analysis**.

Tool	Type	Target	Features^a^	Website	Reference
MetaboAnalyst2	Web	MS and NMR	1–7	http://www.metaboanalyst.ca/	Xia et al. (2012)
XCMS	R	MS	1–3	http://metlin.scripps.edu/xcms/	Smith et al. (2006)
MetSign	MatLab	MS	1–3	http://metaopen.sourceforge.net/	Lommen and Kools (2012)
XCMS online	Web	LC-MS	1–4	https://xcmsonline.scripps.edu/	Tautenhahn et al. (2012b)
MAVEN	Application	LC-MS	1–7	http://genomics-pubs.princeton.edu/mzroll	Melamud et al. (2010)
mzMine2	Application	LC-MS	1–5	http://mzmine.sourceforge.net/	Pluskal et al. (2010)
MAIT	R	LC-MS	1–5	http://b2slab.upc.edu/software-and-downloads	Fernández-Albert et al. (2014)
OpenMS	Application	LC-MS	1–3	http://open-ms.sourceforge.net/	Sturm et al. (2008)
Metabolome express	Web	GC-MS	1–5	https://www.metabolome-express.org/	Carroll et al. (2010)
Metabolite detector	Application	GC-MS	1–4	http://md.tu-bs.de/	Hiller et al. (2009)
MetDAT	Web	MS	1–5	http://smbl.nus.edu.sg/METDAT2/	Biswas et al. (2010)
FOCUS	MatLab	NMR	1–4	http://www.urr.cat/FOCUS/	Alonso et al. (2013)
Automics	Application	NMR	1–2, 5	https://code.google.com/p/automics/	Wang et al. (2009)
Bayesil	Web	NMR	1–4	http://bayesil.ca/	Ravanbakhsh et al. (2014)
Speaq	Application	NMR	1–2, 5	https://code.google.com/p/speaq/	Vu et al. (2011)
MetaboLab	Application	NMR	1–2, 5	http://www.nmrlab.org.uk/	Ludwig and Gunther (2011)
rNMR	R	NMR	8	http://rnmr.nmrfam.wisc.edu/	Lewis et al. (2009)
MetaboMiner	Application	NMR	8	http://wishart.biology.ualberta.ca/metabominer/	Xia et al. (2008)
Muma	R	–	5	http://cran.r-project.org/web/packages/muma	Gaude et al. (2013)
MetaXCMS	R	MS and NMR	5	http://metlin.scripps.edu/metaxcms/	Tautenhahn et al. (2010)
BATMAN	R	NMR	3–4	http://batman.r-forge.r-project.org/	Hao et al. (2012)
AStream	R	LC-MS	4	http://www.urr.cat/AStream/AStream.html	Alonso et al. (2011)
Camera	R	LC-MS	4	http://metlin.scripps.edu/xcms/	Kuhl et al. (2011)
MetaboHunter	Web	NMR	4	http://www.nrcbioinformatics.ca/metabohunter/	Tulpan et al. (2011)
MetScape	Application	–	6–7	http://metscape.ncibi.org/	Gao et al. (2010)
IMPaLA	Web	–	6–7	http://impala.molgen.mpg.de/	Kamburov et al. (2011)
MetExplore	Web	–	6–7	http://metexplore.toulouse.inra.fr/	Cottret et al. (2010)
MetPA	Web	–	6–7	http://metpa.metabolomics.ca/	Xia and Wishart (2010a)
Cytoscape	Application	–	7	http://www.cytoscape.org/	Smoot et al. (2011)
Vanted	Application	–	7	http://vanted.ipk-gatersleben.de/	Rohn et al. (2012)
Paintomics	Web	–	7	http://www.paintomics.org/	García-Alcalde et al. (2011)

*This table provides a complete and updated list of the open-source software that is commonly used in the untargeted analysis of metabolomic data*.

*^a^This column refers to the features included in the tool: spectral pre-processing (1), spectral/peak alignment (2), peak detection (3), metabolite identification (4), data analysis (5), pathway analysis (6), pathway visualization (7), and 2D-NMR analysis (8)*.

## Spectral Processing

**Figure 3:**
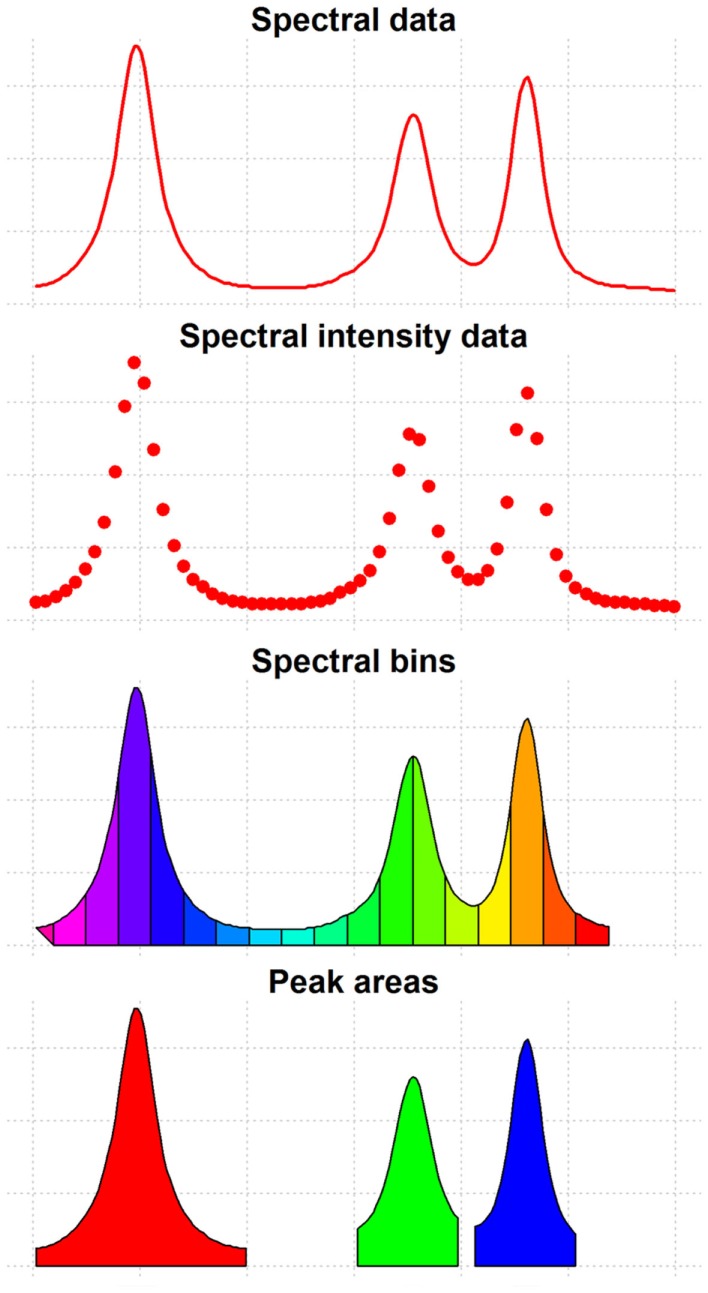
**Features of spectral data**. This figure shows the different types of features that can be extracted from spectral data and used for data analysis.

Spectral processing is a methodological approach aimed at accurately identifying and quantifying the features in the sample spectra of a metabolomics study (Figure 3). Metabolomic spectra are sequentially or jointly processed until a final set of feature quantifications is obtained. Spectral processing is also necessary to guarantee that each final measurement will refer to the same metabolomic feature in all samples. The data resulting from spectral processing is generally arranged in a feature quantification matrix (FQM) that contains the quantification of the metabolic features of all the analyzed samples and that will be used as input for subsequent statistical analysis.

### Spectral pre-processing

In order to improve the signal quality and reduce possible biases present in the raw data, several pre-processing steps are usually applied. In NMR- and MS-based spectra, baseline correction is used to remove low frequency artifacts and differences between samples that are generated by experimental and instrumental variation (Dietrich et al., 1991; Smith et al., 2006; Xi and Rocke, 2008; Zhang et al., 2010). After this, the application of high-frequency filters may be necessary to remove the electronic noise present in the data that is generated by the measurement equipment.

### Feature-detection

The objective of the feature-detection step is to identify and quantify the features present in the spectra. Peak-based methods are the most common algorithmic choice for feature-detection in MS-based studies (Gika et al., 2014; Niu et al., 2014; Rafiei and Sleno, 2015). These methods detect the peaks across the spectrum and integrate their areas to provide a quantification of the underlying metabolite. In this approach, spectral alignment is also generally applied either before or after peak detection. In NMR studies, binning-based approaches have been commonly used to detect feature peaks in complex biological samples. However, these methods perform poorly compared to peak-based methods, particularly in those cases where there is significant spectral unalignment, or in those cases where multiple peaks from different metabolites are captured by the same spectral bin (Vu and Laukens, 2013). For these reasons, peak-based methods are increasingly being used in NMR-based studies (Wishart, 2008). Nonetheless, there have been recent developments in binning algorithms, particularly in the detection of the optimal binning boundaries that have improved the performance of this feature-detection approach (Sousa et al., 2013).

Peak overlap is also a common problem in NMR-based studies. Overlapping peaks are treated as one same feature both in binning and peak-based approaches. Consequently, the results obtained from the analysis of these variables can be often hard to interpret. To attempt to solve this problem, spectral deconvolution methods have been developed (Hao et al., 2014). These methods, which are based on the fitting to metabolite spectral templates, are able to extract independent metabolite quantifications from a set of overlapping peaks. The main disadvantage of this type of algorithms, however, is that they depend on the existence of spectral libraries of each metabolite and, therefore, they are unable to quantify peaks arising from previously uncharacterized metabolites.

#### Peak detection

The most commonly used peak detection algorithms analyze each sample spectrum independently (Tautenhahn et al., 2008, 2012b; Pluskal et al., 2010). These methods are based on two analytical steps (Yang et al., 2009). In the first step, the spectra are smoothed. For this objective, multiple different filters are available (i.e., moving average, Gaussian, Savitzky-Golay…; Yang et al., 2009). From these, however, the Wavelet transform-based filters have demonstrated a superior performance, although at the expense of a higher computation time (Du et al., 2006; Tautenhahn et al., 2008). This performance improvement is mainly due to the ability of the Wavelet transform to work with the unequal peak widths that characterize metabolomic spectra. In the second step, the different metabolite peaks are identified using one or multiple detection thresholds. These thresholds are applied to different parameters such as the signal-to-noise ratio, the intensity, or the area of each peak from the resulting filtered spectra (Yang et al., 2009). In metabolomics studies involving large numbers of samples, a frequency filter (i.e., consensus peak signal), can be also applied so that only those peaks that are present in a minimum percentage of samples are selected for downstream analysis.

#### Spectral alignment

Spectral alignment is one of the main processing steps in metabolomic studies involving multiple samples. When analyzing multiple spectra, the position of the peaks corresponding to the same metabolic feature may be affected by non-linear shifts. In NMR-based studies, these shifts are observed in the ppm axis and are usually introduced by differences in the chemical environment of the sample like ionic strength, pH, or protein content (Weljie et al., 2006; Xiao et al., 2009). In MS-based studies, peak shifts are mainly observed across the retention time axis, and are generally associated with changes in the stationary phase of the chromatographic column (Burton et al., 2008). Spectral alignment methods must be therefore applied to correct this undesired variability in the samples that can profoundly affect the quality of the study. Spectral alignment algorithms can be divided in two main groups: (i) spectral alignment methods, where the spectral data is aligned before peak detection and (ii) peak-based alignment methods, where spectral peaks are aligned across samples once they have been detected using their coordinates (ppm in NMR, and m/z and retention time in LC/GC-MS).

Spectral alignment methods are classified into warping and segmenting methods. Warping methods are based on the application of a non-linear transformation to the ppm (in NMR spectra) or the retention time (in LC/GC-MS) axis in order to maximize the correlation between the spectra. The alignment is then performed by either stretching or shrinking spectral segments to reach this correlation maximization. Among these methods, correlation optimized warping (COW) and dynamic time warping (DTW) are the most commonly used. COW is a segmental alignment method that aligns one sample spectrum toward a reference spectrum (Tomasi et al., 2004). This is done by splitting the original sample and reference spectra into small segments, and by separately aligning each pair of segments. Alignment is performed through dynamic programing in such a way that limited changes in segment lengths are allowed. This way, the overall correlation between both spectra is effectively maximized. In the particular case of crowded spectral regions with large peak shifts, COW has demonstrated to perform particularly well compared to other methods. An alternative to COW method, DTW is a spectral alignment method (Tomasi et al., 2004) that is also based on dynamic programing, and where a warping path is computed to which the connected data points of each spectrum are equivalent. During this last decade, other warping approaches have been developed (Eilers, 2003; Forshed et al., 2003; Lee and Woodruff, 2004; Clifford et al., 2009).

Spectral segmenting methods differ from spectral warping methods in that alignment is performed by applying a constant shift to all the spectral points. These methods either align the overall spectra or split the spectra into smaller segments and independently align each resulting segment. The Icoshift algorithm (Savorani et al., 2010) is one of the most commonly used segmentation methods, and is based on the convergence toward a reference signal. This convergence is performed by applying shifts that maximize the segment spectral correlation, which is normally computed using the fast Fourier transform (FFT) to speed up the required calculations (Wong et al., 2005). Icoshift and other correlation-based methods can also be combined with automatic segmentation methods (Veselkov et al., 2008), which are able to optimally split the spectra in order to improve the alignment of the resulting spectral segments. However, the use of a reference spectrum has several disadvantages. Very recently, the RUNAS algorithm implemented in the FOCUS processing workflow (Alonso et al., 2013) has provided a spectral segmenting method that avoids the use of a reference spectrum. Instead, the FOCUS method uses the information from the different sample spectra to iteratively maximize the inter-sample weighted-mean correlation. This approach has shown that avoiding the use of a reference spectrum is a powerful strategy to avoid many of the analytical biases derived from its use. These biases are mainly due to the fact that the reference spectrum may not be representative of the spectral diversity present in the samples. FOCUS alignment algorithm has also shown that an appropriate spectral transformation prior to alignment avoids the biases due to the presence of multiple peaks in the same alignment window. Under these conditions, the methods based in correlation maximization without prior transformation are more prone to align the most relevant peak of each sample regardless of whether they correspond to the same metabolic feature or not.

Fast Fourier transform-based segmenting methods such as RAFFT, Icoshift, and FOCUS not only are able to process large metabolomics datasets in a reduced amount of time, but also have shown to perform better than spectral warping methods (Giskeødegård et al., 2010; Savorani et al., 2010; Alonso et al., 2013; Jiang et al., 2013). Within the different segmenting methods, reference-free methods avoid the biases introduced by using reference spectra, but at a cost of being more computationally intensive.

Of relevance, the results reported by several performance comparison studies using either NMR or MS have demonstrated that spectral alignment algorithms have a good performance irrespective of the analytical technique that has been used (MS or NMR; Van Nederkassel et al., 2006; Giskeødegård et al., 2010). Consequently, methods that were initially developed to align NMR spectra are also applied to align MS spectra and vice versa.

Compared to the warping and segmentation alignment methods, peak-based methods are applied after peak detection. In these methods, peak coordinates are used to perform the alignment. This type of method is implemented in the XCMS software (Tautenhahn et al., 2012b), one of the most commonly used methods to process data from LC-MS studies. Given that the shifts along the m/z axis are minimal and the m/z axis has a high resolution, the data can be safely binned in m/z intervals, and peak alignment performed on each bin along the chromatographic time. The XCMS algorithm computes the retention time boundaries within which the observed peaks are expected to represent the same metabolomic feature across the different samples. The computation of these retention time boundaries is performed by using a kernel density estimator. Another common alignment method used in MS is the RANSAC algorithm (Pluskal et al., 2010). In this approach, the corresponding peaks across samples are identified by using a LOESS regression on different retention times and m/z windows.

### Feature normalization

In order to perform an accurate quantification of the features in a metabolomic analysis, a data normalization step is generally required. The objective of normalization is to remove undesired systematic biases, so that only biologically relevant differences are present in the data. This procedure is crucial when analyzing complex biofluids like blood, where the differences in metabolite concentration between samples can be high, and the introduction of internal standards is complicated. Although multiple statistical models have been developed for this objective (Craig et al., 2006; Kohl et al., 2012), the two perhaps most commonly used methods are the use of endogenous stable metabolites (like creatinine in urine) and the use of the total spectral area [i.e., area under the curve (AUC); Weljie et al., 2006; Rasmussen et al., 2011].

### Deconvolution methods in targeted analysis

**Figure 4:**
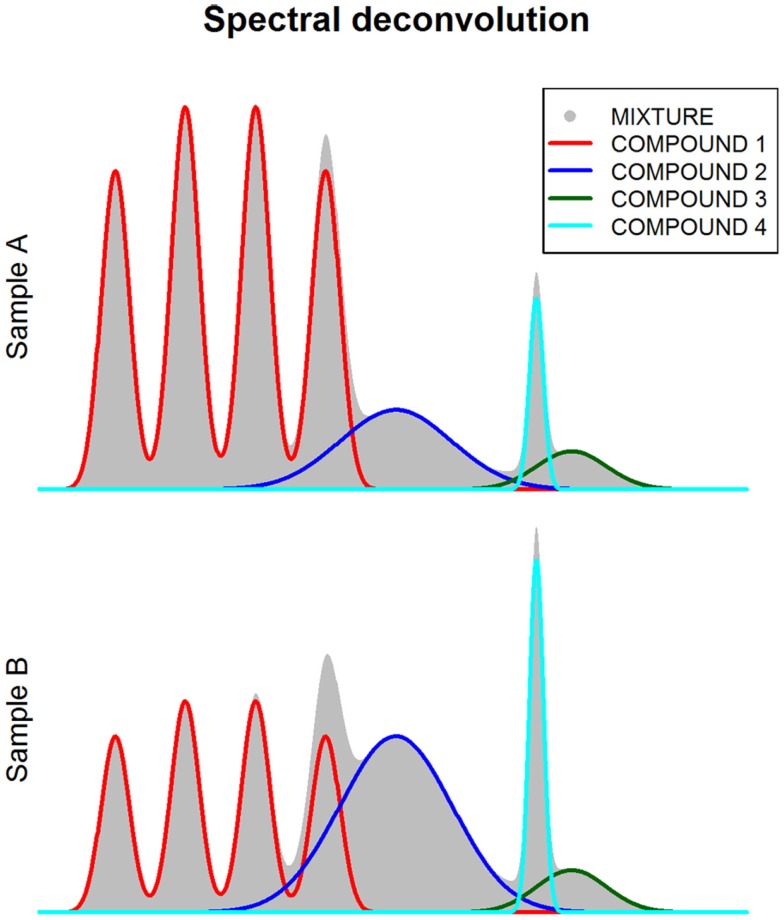
**Spectral deconvolution**. This figure shows how spectra (gray shaded area) can be decomposed (i.e., deconvoluted) in multiple components corresponding to different metabolite compounds.

One of the main limitations for the quantification of metabolomic features is the overlap between peaks from different metabolites. NMR and GC-MS spectra are particularly prone to this type of bias. In order to deal with this technical issue, several methodological approaches have been developed. These approaches are based on spectral deconvolution (Chylla et al., 2011; Astle et al., 2012; Du and Zeisel, 2013; Hao et al., 2014), a signal processing technique that estimates the relative area corresponding to each individual peak when multiple peaks overlap within the same spectral region (Figure 4). However, an important limitation of deconvolution methods is that prior knowledge of the compounds present in the mixture is required. Additionally, the use of these methods in untargeted metabolite studies is yet not possible due to computational intractability (Hao et al., 2014).

The usual input data for these methods is the spectral data from the study and at template library containing the reference peak patterns of each metabolite. Currently, there are multiple methods available for spectral deconvolution of NMR data (Chylla et al., 2011; Zheng et al., 2011; Astle et al., 2012; Hao et al., 2014) and they are mostly based on Bayesian model selection. Among them, BATMAN (Hao et al., 2012) is one of the most frequently used, providing a rich and user-friendly interface and a complete protocol to perform this type of analysis (Hao et al., 2014). BATMAN is an open-source software and its performance has been demonstrated to be very similar to that of the NMR Suite software package (Chenomx Inc., Edmonton, AB, Canada; Weljie et al., 2006), a proprietary software that is considered a gold standard for NMR metabolomics (Chenomx Inc., Edmonton, AB, Canada; Weljie et al., 2006). The NMR Suite itself provides a semi-automated tool for spectral deconvolution which allows interactive fitting of the metabolite peaks to the reference metabolite spectra. The major disadvantage of this tool is the large amount of time required to process large sample datasets and the need of highly skilled data analysis specialists.

GC-MS methods for spectral deconvolution are mostly based on unsupervised approaches that do not require the prior knowledge of the compounds presents in the sample (Stein, 1999; Hiller et al., 2009; Ni et al., 2012). These approaches are mainly based on three steps, namely: (a) noise analysis for selecting the spectral segments to be deconvoluted, (b) component perception for identification of the individual components present in each segment, and (c) deconvolution for fitting the individual components to the overall spectral shape. Du et al. provide an extensive review of these methods (Du and Zeisel, 2013).

## Data Analysis

Once the metabolite features are robustly quantified, there are multiple univariate and multivariate statistical methods that can be used to perform the desired study analysis. These groups of techniques are commonly known as chemometric methods (Madsen et al., 2010) and usually require some degree of expertise to be conveniently applied. In the following sections, we define the most commonly used metabolomic features, and we describe the most commonly used chemometric methods.

### Metabolomic features

After applying the adequate pre-processing methods, metabolomics data is usually reduced to a FQM. In this data representation, rows correspond to the samples and columns correspond to the different metabolomic features. Each metabolomic feature is intrinsically related to the concentration of a particular metabolite. Depending on the analytical technique and the spectral processing workflow that have been used, different metabolomic features are used as input for data analysis (Figure 3):

- Spectral peak areas: one of the most commonly used features in high-throughput metabolomics data (NMR-based or MS-based). They are computed through the integration of the peaks identified and aligned using the methods described in the previous section (see Spectral Pre-Processing to Deconvolution Methods in Targeted Analysis). Once this data has been analyzed, the identification of the metabolites representing the relevant peaks is required in order to provide biological meaning to the results. Metabolite-identification methods are reviewed in Section “Metabolite Identification and Spectral Databases.”
- Metabolite concentrations: in contrast to the previous features, metabolite identification can be performed prior to data analysis in order to obtain absolute or relative metabolite concentrations to be used as input for data analysis (Wishart, 2008; Zhou et al., 2012). This type of features allow both to reduce the high redundancy of peak areas (i.e., one metabolite is often represented by multiple spectral peaks), and to provide biological significance to all the analyzed features. The most common metabolite-identification methods are reviewed in Section “Metabolite Identification and Spectral Databases.”
- Spectral bin areas: in addition to peak areas and metabolite concentrations, spectral bins (or also buckets) are also commonly used features in NMR-based studies. This technique consists of dividing the spectra into evenly spaced regions that are later integrated to obtain the corresponding spectral bin areas. In order to mitigate problems such as peaks lying in two consecutive integration regions, some methods have implemented uneven binning algorithms like dynamic adaptative binning (Anderson et al., 2011), Gaussian binning (Anderson et al., 2008), and adaptative intelligent binning (De Meyer et al., 2008). This feature estimation approach has, however, some inherent disadvantages produced by the presence of uninformative features in the spectra (i.e., spectral areas without spectral peaks) and the lack of inter-sample feature correspondence when spectra are heavily affected by unalignment (e.g., urine samples with large pH variability).

### Univariate analysis methods

Univariate methods analyze metabolomic features independently. They are common statistical analysis approaches and, therefore, their main advantage is their ease of use and interpretation. However, their main disadvantage is that they do not take into account the presence of interactions between the different metabolic features. The metabolomic data obtained from biological samples is often very complex with the presence of correlations between features from the same metabolite and correlations between metabolites from the same pathway. Also, the effect of potential confounding variables like gender, diet, or body mass index is not taken into account by these analysis methods, increasing the probability of obtaining false positive or false negative results (Winnike et al., 2009; Rasmussen et al., 2011; Townsend et al., 2013).

Several univariate analysis methods are available for metabolomic data analysis. The selection of the method will depend on the statistical properties of the feature distribution (Broadhurst and Kell, 2006; Vinaixa et al., 2012). For example, when assessing differences between two or more groups, parametric tests such as Student’s *t*-test and ANOVA are commonly applied, provided that normality assumptions are conveniently verified. The latter can be confirmed using the Kolmogorov–Smirnov normality test or Bartlett’s homogeneity of variances test. In those cases where normality of the data cannot be assumed, non-parametric tests such as Mann–Whitney *U* test or Kruskal–Wallis one-way analysis of variance are preferable.

In addition to choose the most appropriate statistical analysis test, another important consideration in metabolomic data analysis is the multiple testing problem. In most metabolomic studies, a large number of metabolomic features are analyzed simultaneously and, therefore, the probability of finding a statistically significant result by chance (i.e., false positive) is high. In order to control for this multiple testing issue, several correction methods are available. Each method is characterized by a particular balance between avoiding false metabolite associations (i.e., false positives) and prevents discarding true associations (i.e., false negatives). Depending on the study design, researchers might decide to use a more or less conservative approach. The Bonferroni correction is perhaps the most conservative multiple test correction approach, where the number of type I errors (false positives) regarding to the total number of hypotheses tested [i.e., defined as familywise error rate (FWER)] is minimized at the expense of increasing type II errors (false negatives). In the Bonferroni correction, the significance level for one hypothesis (i.e., alpha value), is divided by the number of hypotheses tested simultaneously. Although a very conservative approach, especially when the hypotheses tested are not independent, many researchers advocate its use in metabolomic studies (Broadhurst and Kell, 2006). Recently, Chadeau-Hyam et al. assessed the metabolome-wide significance level (MWSL) for biomarker identification in urine using a permutation-based method to estimate the correct FWER (Chadeau-Hyam et al., 2010). Their method took into account metabolite collinearity and reported that a conservative estimate of the independent number of tests is 35% of the performed tests. This result indicates that the Bonferroni multiple test correction method might be over conservative.

Other less conservative multiple test correction methods are however available and are mostly based on the minimization of the false-discovery rate (FDR; Benjamini and Hochberg, 1995). While Bonferroni and other FWER-based methods minimize the probability of at least one false positive in the overall set of tests, FDR-based methods minimize the expected proportion of false positives on the total number of positives (Van Den Oord, 2008). Most of these methods have been extensively used for the analysis of gene-expression microarray data, where thousands of genes are tested in parallel (Reiner et al., 2003; Jung, 2005; Xie et al., 2005). In untargeted metabolomic studies, where large numbers of metabolites are simultaneously analyzed, and where it is also expected that more than one or two of these biomarkers will be associated, the use of less strict multiple correction methods like FDR methods might be more useful.

### Multivariate analysis methods

In contrast to univariate methods, multivariate analysis methods take into account all the metabolomic features simultaneously and, consequently, they can identify relationship patterns between them. These pattern-recognition methods can be classified into two groups: supervised and unsupervised methods. In unsupervised analysis methods, the similarity patterns within the data are identified without taking into account the type or class of the study samples. In supervised methods, the sample labels are used in order to identify those features or features combinations that are more associated with a phenotype of interest. Supervised methods are also the basis for building prediction models.

#### Unsupervised methods

Unsupervised methods are often applied to summarize the complex metabolomic data. They provide an effective way to detect data patterns that are correlated with experimental and/or biological variables. Principal component analysis (PCA) is the most commonly used unsupervised method in metabolomic studies (Wold et al., 1987; Bro and Smilde, 2014). PCA is based on the linear transformation of the metabolic features into a set of linearly uncorrelated (i.e., orthogonal) variables known as principal components. This decomposition method maximizes the variance explained by the first component while the subsequent components explain increasingly reduced amounts of variance. At the same time, PCA minimizes the covariance between these components (i.e., they are independent of each other). After applying the PCA method, a set of loading vectors and score vectors are obtained. The loading vectors represent the principal components, and each vector coefficient corresponds to the individual contribution of each variable to the principal component. The score vectors represent the projection of each sample onto the new orthogonal basis. Plotting these sample scores over the first principal components is a convenient way of summarizing the global dataset, since normally these first principal components capture most of the variability in the dataset. PCA is also used in metabolomics studies to assess data quality, since it can identify sample outliers or reveal hidden biases in the study. For example, PCA has been used in several studies to determine the impact of technical variation in the analysis of metabolic profiles (Gika et al., 2008; Winnike et al., 2009; Rasmussen et al., 2011; Yin et al., 2013).

Other unsupervised methods like hierarchical clustering analysis (HCA) and self-organizing maps (SOMs) have also been applied to metabolomic data. These methods can be particularly suitable to detect non-linear trends in the data that are not conveniently covered by PCA. SOMs have been used in metabolomics studies to visualize metabolic phenotypes and feature patterns as well as to prioritize the metabolites of interest based on their similarity (Kohonen et al., 2000; Meinicke et al., 2008; Mäkinen et al., 2008; Goodwin et al., 2014). HCA is also a powerful clustering and visualization tool that provides a clustering procedure at the feature and sample levels according to a predefined distance measure (Brauer et al., 2006; Sreekumar et al., 2009).

#### Supervised methods

Supervised methods are used to identify metabolic patterns that are correlated with the phenotypic variable of interest while down-weighting the other sources of variance. These methods are also the basis for building classifiers based on metabolomic features (Xia et al., 2013). Partial least squares (PLS; Fonville et al., 2010) is one of the most widely used supervised method in metabolomics. It can be used either as a regression analysis (i.e., quantitative variable of interest) or as a binary classifier (PLS-DA; i.e., binary variable of interest). Unlike PCA, PLS components do not maximize the explained dataset variance but the covariance between the variable of interest and the metabolomic data. Therefore, the feature coefficients (loadings) of PLS components represent a measure of how much a feature contributes to the discrimination of the different sample groups. However, one weakness of PLS is that some metabolic features that are not correlated with the variable of interest can influence the results. In order to deal with this problem, orthogonal PLS (O-PLS; Trygg and Wold, 2002) were developed. O-PLS models evolved from PLS models and factorize the data variance into two components: a first component which is correlated with the variable of interest and a second uncorrelated component (i.e., orthogonal). Classification of metabolomics samples is commonly performed by fitting the discriminant analysis versions of PLS and O-PLS models (i.e., PLS-DA, O-PLS-DA; Kemsley, 1996; Bylesjö et al., 2006).

The performance of PLS and O-PLS models has been extensively compared but, to date, there is no agreement as to which of the two methods is superior (Tapp and Kemsley, 2009). In the last years, however, a progressive move from the use of PLS models to O-PLS models has been observed in the metabolomics field (Fonville et al., 2010).

Support vector machines (SVMs) are another class of supervised analysis methods to build classifiers based on metabolomic data (Mahadevan et al., 2008; Kim et al., 2010; Luts et al., 2010). Although classifiers based on SVM are harder to interpret, they are able to manage the presence of non-linear relations between the metabolomic data and the variable of interest.

#### Multiway methods for longitudinal metabolomic data

There is also a wide range of methods that are designed to provide a comprehensive interpretation of the metabolic changes according to the organization of the analyzed samples (i.e., samples from different tissues or corresponding to time series in a longitudinal study). These methods decompose the original multiway (i.e., multi-dimensional) data matrix into a set of easily interpretable factors. In NMR studies, two of the most commonly used methods are parallel factor analysis (PARAFAC) and multivariate curve resolution (MCR). The input data for these methods is commonly a three dimensional (3D) matrix with coefficients *c_ijk_* (where *i* represents a metabolic feature, *j* the analyzed individual, and *k* the tissue from which the sample was extracted or the sample extraction time-point). The PARAFAC analysis of a 3D matrix generates three loading matrices that capture the contributions of each metabolic feature, of each individual, and of each tissue type or time-point. Alternatively, MCR analysis decomposes the 3D matrix into a set of two factors which contain the contributions of each metabolic feature and each analyzed sample. To do this, the 3D matrix must be fitted in a 2D matrix, where the different metabolic features are arranged on the first dimension while the each individual and tissue/time-point are arranged on the second dimension (Peré-Trepat et al., 2007; Karakach et al., 2009; Montoliu et al., 2009; Martin et al., 2010).

## Biomarker Discovery in Metabolomics

One of the most promising applications of metabolomics in the medical sciences is the identification of biomarkers. New metabolomic biomarkers are usually determined using supervised analysis models since they are capable to aggregate the evidence of multiple metabolites. The usefulness of the resulting classification models must be then evaluated in order to consider their use in real clinical settings. Performance assessment and model validation are crucial analytical steps for the evaluation of metabolomic classification models.

### Performance assessment

Performance assessment measures how well the outcome predicted by our model matches the real outcome. Several complementary measures are available to assess the classifier performance: predictive accuracy (percentage of correctly classified subjects), sensitivity (percentage of true positives that are correctly classified), and specificity (percentage of true negatives that are correctly classified). These three measures allow the assessment of the classifier performance given a fixed decision boundary. However, these performance measures tend to be dependent on the outcome prevalence and on the decision boundary chosen (Xia et al., 2013). The receiver operating characteristic (ROC) curve avoids this type of bias and is the most used performance assessment method. ROC curve estimation is a non-parametric procedure consisting of the comparison of specificity against sensitivity according to a specific decision boundary. ROC curves are often summarized by the AUC metric. The AUC metric gives the probability that a classifier will rank a randomly chosen positive sample higher than a randomly chosen negative one. Therefore, a perfect classifier will obtain AUC = 1 while a random classifier will obtain AUC close to 0.5. An AUC >0.7 is often considered the minimal performance for a biomarker test to be considered clinically useful (Xia et al., 2013). In addition to the overall performance assessment using the AUC metric, the ROC curves can also be used to determine the optimal decision boundary for the classifier (Xia et al., 2013). ROC curve estimation is a common analysis and therefore, multiple tools are available for ROC-based performance evaluation like the R packages ROCR (Sing et al., 2005) and pROC (Robin et al., 2011), as well as the ROCCET (Xia et al., 2013) web application.

### Model validation

When designing classification models, a validation step is required to estimate how well the classification model will perform when applied to new samples. This step is particularly important when using small sample sizes in order to discard model overfitting. Two main approaches are available for performing this task: permutation testing and cross-validation (Westerhuis et al., 2008).

The aim of the permutation-based validation is to measure the performance of the predictor model by determining the probability of observing an equal or better performance by pure chance. This analysis is performed by estimating the null distribution of the performance measures (i.e., AUC) under the assumption that no differences exist between sample groups. This is done by randomly permuting multiple times the sample group classes (e.g., case-control) and calculating the statistic under each permuted dataset. Once computed, the performance measures of the *true* model (i.e., based on the real sample status) should lie outside the chosen confidence intervals (e.g., 95 or 99%) of the estimated null distributions in order to be considered significant. In contrast with the permutation approach, cross-validation approaches estimate the predictive performance of a classifier using an iterative approach. At each round of cross-validation, the total sample is split into a training group and a testing group. In the former group, the predictor model is built using a specific set of parameters. The performance of this model is then evaluated using the remaining group of samples. This procedure is repeated several times so that all the samples have been used once as a testing group. Averaging these results we will obtain an unbiased estimate of the performance of the predictor. The size of the testing sample can be composed by several samples (i.e., *n*-fold cross-validation) or can be as small as a single individual (i.e., leave-one out cross-validation). This approach provides a good measure of how data overfitting affects to the computed model. When the used models require optimization (i.e., optimal number of PLS/O-PLS components to be used) a double cross-validation schema is usually required: a first cross-validation step is applied to optimize the model and a second step for assessing the model quality (Westerhuis et al., 2008; Szymanska et al., 2012). The double cross-validation schema requires the dataset to be iteratively split in two sets S1 and S2. In the first step, the S1 set is randomly divided into two subsets S11 and S12, where S11 is used to compute models with different number of components and the S12 set is used to evaluate the prediction power of each model. This procedure is repeated until all the samples in S1 have been once in the S12 set, and the model with the lowest prediction error is selected. In the second step, the S2 set is used to assess the performance of the optimal model as computed in step one. This global analysis is performed recursively by randomly splitting the global dataset in sets S1 and S2 until all the samples have been once in S1. Further details on the different types of cross-validations are described in more detail elsewhere (Westerhuis et al., 2008; Szymanska et al., 2012).

## Metabolite Identification and Spectral Databases

Metabolite identification is one of the major challenges of high-throughput metabolomic analysis. This step is indispensable to confer a biological meaning to the associated features in a metabolomic study. In MS-based studies, the common metabolite-identification approach is based on querying metabolomic databases for the neutral molecular mass values of the identified peaks using a tolerance window. The neutral molecular mass is inferred from the peak m/z value, and depends on the chemical nature of the identified peak (i.e., ionization mode and ionization adduct). Assuming no prior knowledge, each peak m/z value can lead to multiple plausible neutral molecular masses that can represent different ionization adducts (H^+^, Na^+^, K^+^, …). This multiplicity often results in a high number of false positive identifications. In order to reduce false positives, several methods have been developed. AStream and Camera are methods designed to identify isotopic and adduct patterns in order to reduce data complexity in MS experiments (Alonso et al., 2011; Kuhl et al., 2011). Using these approaches, the chemical nature of each selected ion peak is estimated, and only one neutral mass is inferred from each identified pattern. Using these methods has the added advantage of improving the ascertainment of true biological compounds.

In NMR-based studies, automatic metabolite identification is commonly performed by matching the measured NMR peaks against a set of reference metabolite patterns. Each metabolite reference spectrum is defined by one or multiple peaks, which are characterized by their ppm positions and their relative intensities. MetaboHunter is an online tool for identifying compounds by matching the reference peak positions against the list of detected peak positions (Tulpan et al., 2011). However, this approach can lead to high false positive rates, since it only uses one peak parameter to match reference peaks. The MetaboHunter approach has been superseded by more recent methods based on the valid cluster concept (Mercier et al., 2011; Jacob et al., 2013). In addition to using the ppm position, these methods include peak intensities and inter-sample intensity correlation as parameters for matching data peaks to reference peaks. The NMR analysis workflow implemented in FOCUS follows this same metabolite-identification approach, with the added advantage that it also accounts for the presence of missing peaks generated by spectral overlapping (Alonso et al., 2013).

Metabolite spectral databases are essential for metabolite identification. The quality of the stored data as well as the number of metabolite spectra available in these databases is critical for the performance of identification algorithms. During the last years, multiple databases have been developed (Table 2) and the number of available metabolite reference spectra is continuously growing (Ellinger et al., 2013; Fukushima and Kusano, 2013). The Human Metabolome Database (HMDB) is perhaps the most extensive public metabolomic spectral database to date (Wishart et al., 2013). The HMDB stores >40,000 different metabolite entries, with exhaustive biological metadata and MS/NMR spectral references. In addition to spectral databases, several studies have also contributed to characterize the metabolome of multiple types of samples. Many of these reference studies are also exceptional resources of high quality data associated with the biofluid, tissue, or cell type of interest (Wishart et al., 2008; Psychogios et al., 2011; Bouatra et al., 2013).

**Table 2:** **Spectral databases available for metabolite identification**.

Database	Spectral data	Website	Statistics	Reference
HMDB	MS/NMR	http://www.hmdb.ca	41,806 metabolite entries and 1,579 metabolites with spectra (^1^H-NMR, LC-MS, GC-MS …)	Wishart et al. (2013)
LMSD	MS	http://www.lipidmaps.org	37,500 lipid structures with MS/MS spectra	Sud et al. (2007)
METLIN	MS	http://metlin.scripps.edu	240,516 metabolite entries and 12,057 metabolites with MS/MS spectra	Tautenhahn et al. (2012a)
TOCCATA COLMAR	NMR	http://spin.ccic.ohio-state.edu	Multiple spectral NMR datasets: ^1^H- and ^13^C-NMR, 2D ^13^C–^13^C TOCSY (*n* = 463), 2D ^1^H–^1^H TOCSY and ^13^C–^1^H HSQC-TOCSY (*n* = 475), and 2D ^13^C–^1^H HSQC (*n* = 555)	Robinette et al. (2008), Bingol et al. (2012, 2014, 2015)
MassBank	MS	http://www.massbank.jp	2,337 metabolites and 40,889 spectra (LC-MS, GC-MS …)	Horai et al. (2010)
Golm metabolome	GC-MS	http://gmd.mpimp-golm.mpg.de	2,019 metabolites with GC-MS spectra	Hummel et al. (2007)
BMRB	NMR	http://www.bmrb.wisc.edu	9,841 biomolecules with ^1^H, ^13^C, or ^15^N spectra	Ulrich et al. (2008)
Madison	NMR	http://mmcd.nmrfam.wisc.edu	794 compounds with spectra including ^1^H, ^13^C, ^1^H–^1^H, ^1^H–^13^C …	Cui et al. (2008)
NMRShiftDB	NMR	http://nmrshiftdb.nmr.uni-koeln.de	42,840 structures and 50,897 measured spectra	Steinbeck et al. (2003)
RIKEN	MS/NMR	http://prime.psc.riken.jp	1,589 metabolites (*Arabidopsis*)	Akiyama et al. (2008), Sakurai et al. (2013)
Birmingham Metabolite Library	NMR	http://www.bml-nmr.org	208 metabolites and 3,328 1D- and 2D-NMR spectra	Ludwig et al. (2012)

*This table shows a list of the spectral databases that are most commonly used in current metabolomics studies to characterize the associated metabolite features*.

## Pathway and Network Analysis of Metabolomic Data

Pathway and network analysis approaches increase the information generated by metabolomic studies. Both approaches exploit the relational properties present in metabolomic data. Pathway analysis uses prior biological knowledge to analyze metabolite patterns from an integrative point of view. Alternatively, network analysis uses the high degree of correlation existing in metabolomics data to build metabolic networks that characterize the complex relationships existing in the set of measured metabolites.

### Pathway analysis

Until very recently, when analyzing metabolomic data no prior knowledge regarding metabolite relationships could be assumed. During the last years, however, the biological knowledge available for metabolomics studies has been constantly increasing. Metabolic pathways are groups of metabolites that are related to the same biological process, and that are directly or indirectly connected by one or multiple enzymatic reactions. Biological databases such as Kyoto Encyclopedia of Genes and Genomes (KEGG; Kanehisa et al., 2012), small molecule pathway database (SMPDB; Jewison et al., 2014), EHMN (Ma et al., 2007), WikiPathways (Kelder et al., 2012), and MetaCyc (Caspi et al., 2008) provide exhaustive information of a large number of metabolic pathways (Table 3). The availability of this data is therefore enabling the use of pathway-based approaches in metabolomics. These methods are currently referred as metabolite set enrichment analysis (MSEA), and are methodologically based on the gene set enrichment analysis (GSEA) approach, designed for pathway analysis of gene-expression data (Khatri et al., 2012).

**Table 3:** **Biological databases for pathway analysis**.

Database	Description	Website	Reference
Kyoto Encyclopedia of Genes and Genomes (KEGG)	466 pathways, 17,333 metabolites, and 9,764 biochemical reactions	http://www.genome.jp/kegg/	Kanehisa et al. (2012)
MetaCyc	2260 pathways from 2600 different organisms	http://metacyc.org/	Caspi et al. (2008)
The small molecule pathway database (SMPDB)	1,594 metabolites mapping 727 small molecule pathways found in humans	http://www.smpdb.ca/	Jewison et al. (2014)
WikiPathways	1,910 pathways	http://wikipathways.org/	Kelder et al. (2012)
Plant metabolic network (PMN/PlantCyc)	Multi-species pathway database for plant metabolomics	http://www.plantcyc.org/	Chae et al. (2014)

*This table describes the main databases that provide biological information on metabolites and metabolic pathways*.

To date, three different approaches have been developed to perform MSEA (Xia and Wishart, 2010b):

- Overrepresentation analysis (ORA): Given a list of metabolite pathways or groups of metabolites of interest, a hypergeometric test or a Fisher’s Exact test is used to evaluate whether the metabolites of these groups are represented more than expected by chance. When the input metabolite list is defined as the set of metabolites which are differentially expressed in the analyzed phenotypes, the ORA results may identify metabolic pathways that are globally associated to these phenotypes.
- Quantitative enrichment analysis (QEA): Unlike ORA, the input data for this method is a set of metabolite concentrations from multiple samples. Enriched pathways can be identified using different approaches like globaltest (Goeman et al., 2004), globalAncova (Hummel et al., 2008), or the Wilcoxon-based test (Adjaye et al., 2005). Enriched pathways include pathways where a few number of compounds are significantly changed or pathways where a large number of metabolites are slightly but consistently changed (Xia and Wishart, 2010b).
- Single-sample profiling (SSP): While the two previous methods are suited for studies involving large numbers of samples, this approach can be used at the sample level. The input data for SSP analysis is an input list of normalized metabolite concentrations in a common biofluid, tissue, or cell type and a database with the normal concentration ranges of these metabolites in the sample. From this input data, SSP identifies the set of metabolites showing levels significantly different from the normal concentration ranges.

In order to improve the interpretability of pathway analysis results, MSEA results can be combined with pathway topological measures. These measures allow the assessment of impact of the unbalanced metabolites within the overrepresented pathway. First, single impacts are evaluated using the degree and betweenness network centrality measures of each metabolite (Aittokallio and Schwikowski, 2006). Subsequently, the overall impact (i.e., pathway impact; Xia and Wishart, 2010a) is calculated as the sum of the single impact measures of the unbalanced metabolites normalized by the sum of the impact measures of all the metabolites within the pathway.

Metabolomics researchers currently have a wide variety of software tools to analyze metabolomic data at the pathway level. Applications such as Paintomics (García-Alcalde et al., 2011), Vanted (Rohn et al., 2012), and Cytoscape (Smoot et al., 2011) provide different pathway visualization tools. In these tools, the metabolites are mapped on predefined metabolic pathways, and allow a high level of interaction with the data. In addition to visualization tools, Impala (Kamburov et al., 2011) and MetScape2 (Karnovsky et al., 2012) are software tools that also implement specific MSEA methods. Finally, Metaboanalyst is a highly versatile pathway analysis tool, providing a wide range of MSEA methods as well as topological and visualization tools (Xia et al., 2012).

### Correlation-based network analysis

One of the main features of biologic data is the high level of correlation existing between the different elements (i.e., mRNAs, proteins, metabolites). Part of these relational patterns is due to metabolites that belong to common metabolic pathways. In other cases, however, the observed correlations may be due to other causes like global perturbations (i.e., metabolic compounds showing diurnal variation in time series analysis), specific perturbations (i.e., changes in enzyme concentrations spread through their related metabolic pathways), or the intrinsic variability of metabolomic data (Steuer et al., 2003; Camacho et al., 2005; Steuer, 2006). Consequently, metabolites that do not show significant differences across the studied phenotypes may still show different correlation patterns with other metabolites in each phenotype. These patterns can provide valuable information about the underlying metabolic network associated to a specific biological process (Steuer, 2006).

Unlike pathway analysis, correlation-based methods build metabolite networks according to the relationship patterns observed in the experiment data. In the resulting network, each metabolite is represented by a network node but, in contrast to pathway analysis, the links between nodes represent the level of mathematical correlation between each pair of metabolites. In metabolomics data, high correlation coefficients are frequent due to the presence of systemic and indirect associations (Krumsiek et al., 2011). Using classical correlation coefficients leads to highly crowded networks where direct and indirect associations are not distinguished (Langfelder and Horvath, 2008). This problem can be successfully overcome using partial correlation (Krumsiek et al., 2011; Valcárcel et al., 2011). In this approach, the correlation between two metabolites is conditioned against the correlation with the remaining metabolites. Consequently, partial correlation allows to discriminate between direct and indirect (i.e., mediated by other metabolites) metabolite correlations. Valcárcel et al. used this approach to build two different networks corresponding to individuals with normal fasting glucose and individuals with prediabetes (Valcárcel et al., 2011). Although few differences were found between individual metabolite concentrations, the network analysis performed in this study revealed significant changes in lipoprotein metabolism, which is known to be associated with diabetes pathophysiology. Netzer et al. used a similar approach to identify highly discriminant metabolites between healthy controls and individuals with obesity (Netzer et al., 2012). In this case, the metabolic network was built using Pearson’s correlation coefficient, and the differential metabolites were evaluated by using different network descriptors. In the same study, Netzer et al. used the metabolic differences between two sample groups to build a metabolite ratio network (Netzer et al., 2011). In this approach, the link between two metabolites is scored according to the differences in the ratios between the corresponding metabolites in the two sample groups. The resulting network topology is then based on the metabolic differences between the two studied phenotypes. Recently, Kotze et al. have extended the correlation-based network approach to include prior biological knowledge (Kotze et al., 2013). In this approach, the resulting network is mapped onto known metabolic pathways in order to identify novel links within the metabolic network that may play a key role in the phenotypic trait being studied.

## Integration of Omics Data

Systems biology is the computational modeling of complex biological systems at different molecular levels through the analysis of high-throughput data. Systems biology methods can therefore improve our understanding of the biological processes that are associated with a certain phenotype. These approaches also allow studying how the dysregulation of specific biological pathways is propagated across the biological system. The characterization of the complex and often noisy biological systems has become a major challenge in bioinformatics.

### Metabolomics integration with whole genome variation

The association between genome-wide genetic variation and high-throughput metabolomic data is one of the current main objectives of omics data integration. The joint analysis of both types of biological data, known as metabolite genome-wide association studies (mGWAS), has allowed the identification of a large number of genomic regions associated with metabolite levels (Gieger et al., 2008; Illig et al., 2010; Suhre et al., 2011a,b; Table 4). These associations are commonly called genetically influenced metabotypes (GIMs), and could play an important role in the heritability of phenotypic traits. The association between genetic variants and phenotypic traits that often show small association effect sizes can be significantly increased when using intermediate phenotypes like metabolite concentrations (Gieger et al., 2008). These intermediate phenotypes (or endophenotypes) may be characterized by larger effect size associations since they are continuous variables that reflect the actual state of the biological system.

**Table 4:** **List of studies integrating genomics and metabolomics data**.

Cohort size^a^	Metabolites	Biofluid	Metabolomics platform	Objectives	Reference
284	363/40401	Serum	ESI-MS/MS	Study of GIMs	Gieger et al. (2008)
4400	33	Plasma	ESI-MS/MS	Study of GIMs	Hicks et al. (2009)
1809/422	163	Serum	ESI-MS/MS	Study of GIMs	Illig et al. (2010)
1814	163	Serum	ESI-MS/MS	Study of GIMs	Kolz et al. (2009)
862/2031	59	Urine	NMR	Study of GIMs	Suhre et al. (2011b)
1768/1052	276	Serum	UHPLC/MS/MS2, GC/MS	Study of GIMs and overlap with loci of biomedical and pharmaceutical interest	Suhre et al. (2011a)
211	526	Urine and plasma	Multi-platform	Study of GIMs and decomposition of biological population variation in metabolic traits	Nicholson et al. (2011)
4034	153	Plasma	ESI-MS/MS	Study of GIMs and pathway analysis	Demirkan et al. (2012)
8330	216	Serum	NMR	Study of GIMs and heritability of metabolic traits	Kettunen et al. (2012)
6600	130	Serum	NMR	Study of metabolic associations with atherosclerosis using metabolic networks	Inouye et al. (2012)
2076	217	Plasma	HPLC/MS	Study of GIMs and heritability of metabolic traits	Rhee et al. (2013)
7824	486	Plasma	UHPLC/MS/MS2, GC/MS	Study of GIMs, heritability of metabolic traits, and network analysis	Shin et al. (2014)

*This table provides an updated list of studies that have integrated metabolomics data with genomics data*.

*^a^Studies with discovery and validation cohorts are given as *N*_discovery_/*N*_validation_*.

One of the main statistical problems when analyzing the association between genetic variants and metabolite concentrations at a genome-wide level is the large number of tests that must be performed. The number of genetic variants analyzed for each individual by the current high-throughput genotyping technologies usually ranges between 500,000 and 2e^6^. This number of genomic variants can be further increased up to 5–10e^6^ variants with the help of imputation techniques (Howie et al., 2009; Delaneau et al., 2013). Compared to gene-expression data, metabolomic profiles have a much lower number of variables, ranging from 100 s to few 1,000 s. Nevertheless, performing all gene to metabolite association analyses in mGWAS can result in up to 1 ⋅ 10^7^–1 ⋅ 10^11^ statistical tests. To date, there are multiple tools that can efficiently perform this large number of quantitative trait analysis like Matrix eQTL (Shabalin, 2012). However, the main limitation of this type of studies is the number of tests that are performed in parallel, and the associated increase in the false positive rate at the nominal (α = 0.05) level of significance. Applying a conservative multiple test correction methods like the Bonferroni method leads to extremely high significance thresholds (i.e., corrected α levels = 1 ⋅ 10^−9^–1 ⋅ 10^−13^, depending on the total number of performed tests; Gieger et al., 2008; Illig et al., 2010). In order to set a less stringent correction threshold for this type of studies, Demirkan et al. computed the effective number of independent tests by using the number of significant principal components of variation of the metabolomic data (Demirkan et al., 2012). Other studies instead have chosen the genome-wide level of significance commonly used in single-trait GWAS (α = 5e^−8^; McCarthy et al., 2008; Kolz et al., 2009; Tanaka et al., 2009; Rhee et al., 2013).

While most published mGWAS have relied on univariate association tests, there is an increasing effort to develop new multivariate approaches. These approaches have been designed to simultaneously analyze sets of metabolites instead of individual metabolite levels (Klei et al., 2008; Ferreira and Purcell, 2009; O’reilly et al., 2012; Ried et al., 2012; Stephens, 2013). These new approaches have several advantages (Galesloot et al., 2014):

- They take into account the pleiotropic nature of metabolomic data. Subsequently, a genetic variant can be simultaneously associated with multiple metabolites.
- When a genetic variant is associated with multiple metabolites, the aggregated information of the entire set of metabolites increases the statistical power of the analysis (Allison et al., 1998; Zhu and Zhang, 2009).
- By performing a single test for each set of metabolites, the multiple test burden is reduced.

However, one of the main disadvantages of this type of analysis methods is the reduced number of metabolites that can be tested simultaneously. This implies that current metabolite panels (>100 metabolites) cannot be tested together. Inouye et al. overcame this problem by using a two-step design (Inouye et al., 2012). First, using the metabolite correlation matrix they identified the most relevant metabolic networks using hierarchical clustering. The second step consisted of a multivariate GWAS of each selected network. Each genomic variant was therefore tested a much reduced amount of times and, for each test, the loading of each network metabolite was computed.

Pathway-based approaches are also an important approach for the analysis of genetic variation associated with metabolite levels. As described in Section “Correlation-Based Network Analysis,” the methods based on partial correlation coefficients are optimal for the analysis of metabolomic data (Krumsiek et al., 2011). One of these methods, Gaussian Graphical Modeling (GGM), has been recently used to identify unknown metabolites through the integration of metabolomics, GWAS, and pathway data (Krumsiek et al., 2012). Recently, Shin et al. also used GGM to build a complete network of genetic variation associated with human blood metabolite levels (Shin et al., 2014).

### Metabolomics integration with other omics sciences

Recently, the first study analyzing the association of the genome methylation patterns methylation with metabolic traits has been performed (Petersen et al., 2014). In this study, Petersen et al. used multivariate regression analyses to identify two types of methylome–metabotype associations: (a) associations due to underlying genetic variants and (b) independent associations potentially driven by environmental factors influencing the methylome.

In addition to mGWAS studies, several studies have also explored the association between whole genome gene-expression (i.e., transcriptomics) and metabolomics. The data provided by these two omics sciences have been used, for example, to improve the classification of breast cancers and to explore the correlation between the transcriptional and metabolic levels (Borgan et al., 2010). Borgan et al. used the transcriptional data to classify the breast tumor samples according to previously published tumor types. In a second step, they applied hierarchical clustering on each type of samples using the metabolic data. Using this combined approach, new molecular subtypes of tumors were found. Importantly, these new molecular subtypes were better classified than subtypes based only on gene-expression patterns. Additionally, new biological pathways associated with each molecular subtype could be identified. Using GOrilla software (Eden et al., 2009), they were able to identify potential gene groups regulating each analyzed metabolite. Bjerrum et al. recently combined transcriptomics and metabolomics data from colon biopsies of ulcerative colitis patients. They used O-PLS-DA and multivariate logistic regression models to improve the diagnosis of this autoimmune disease (Bjerrum et al., 2014). Zhang et al. also integrated transcriptomics and metabolomics data to study human pancreatic cancer samples (Zhang et al., 2013). Using a correlation-based network analysis, they identified a set of highly co-regulated and decreased metabolites in these samples and subsequently identified the transcripts correlated with these metabolites.

### Toward a complete omics integration

During the last years, high-throughput technologies have enabled the analysis of the biologic variability at multiple molecular levels. The data obtained from the genome, epigenome, transcriptome, proteome, metabolome, or the microbiome can be now combined using systems biology approaches. However, this group of analytical tools is still in its infancy and major improvements in this field will come in the next years (Chen et al., 2012). 3Omics is one good example of this new type of metabolomic analysis tools. 3Omics is one of the first systems biology tools to provide a full integrative analysis including correlation analysis, co-expression profiling, phenotype mapping, pathway enrichment analysis, and GO enrichment analysis at three molecular levels (transcriptome, proteome, and metabolome; Kuo et al., 2013).

## Conclusion

Metabolomics is a research field rapidly evolving to allow the fast and accurate analysis of high-throughput data from diverse biological sources. Although the recent methodologies have been able to overcome several challenges of metabolomics data analysis, there is still much room for improvement. In untargeted metabolomic studies, for example, major improvements are still required in automatic metabolite identification and spectral deconvolution. Although a big effort is being done to improve spectral databases, the development of accurate automatic identification algorithms is still subject to the availability of an exhaustive set of reference metabolite spectra.

In addition to the necessary improvements in the analysis workflow, intense efforts are also being done in the standardization of metabolomics data (Salek et al., 2013a). The Metabolomics Standard Initiative (MSI; Fiehn et al., 2007), currently represents the major community effort to define normalization standards in metabolomics. These developments are based on previous high-throughput data standardization initiatives like MIAME in microarray studies (Brazma et al., 2001), and include the use of minimal reported information, common syntax, data format exchange, and common semantics (Field and Sansone, 2006). To date, general guidelines have been proposed (Sumner et al., 2007) that cover relevant areas in metabolomics studies like biological sample processing, analytical technique details (i.e., instrument description, technique-specific acquisition parameters, and sample preparation), instrumental calibration, validation of the quantification method, data pre-processing, metabolite identification, and nomenclature. Very recently, the MetaboLights database (www.ebi.ac.uk/metabolights) has been launched as a repository to archive and distribute data on metabolomics experiments (Steinbeck et al., 2012; Haug et al., 2013; Salek et al., 2013b). Similar to the established public repositories of transcriptomics data (Barrett et al., 2011), the availability of public repositories for metabolomics data will clearly accelerate the progress in this rapidly evolving field.

Omics sciences like metabolomics are increasing our ability to generate knowledge from multiple aspects of biology. In order to achieve these goals, however, the scientific community will require tools and methods that are able to efficiently integrate all the different sources of molecular and phenotypic information. In the near future, increasingly powerful analysis tools will be developed. The access to these methods in an open-source environment will guarantee its dissemination to the largest scientific community possible.

## Conflict of Interest Statement

The authors declare that the research was conducted in the absence of any commercial or financial relationships that could be construed as a potential conflict of interest.
